# Repurposing Pharmaceuticals Previously Approved by Regulatory Agencies to Medically Counter Injuries Arising Either Early or Late Following Radiation Exposure

**DOI:** 10.3389/fphar.2021.624844

**Published:** 2021-05-10

**Authors:** Vijay K. Singh, Thomas M Seed

**Affiliations:** ^1^ Division of Radioprotectants, Department of Pharmacology and Molecular Therapeutics, F. Edward Hébert School of Medicine, Uniformed Services University of the Health Sciences, Bethesda, MD, United States; ^2^ Armed Forces Radiobiology Research Institute, Uniformed Services University of the Health Sciences, Bethesda, MD, United States; ^3^ Tech Micro Services, Bethesda, MD, United States

**Keywords:** acute radiation syndrome, delayed effects of acute radiation exposure, radiation countermeasures, regulatory agencies, repurposing

## Abstract

The increasing risks of radiological or nuclear attacks or associated accidents have served to renew interest in developing radiation medical countermeasures. The development of prospective countermeasures and the subsequent gain of Food and Drug Administration (FDA) approval are invariably time consuming and expensive processes, especially in terms of generating essential human data. Due to the limited resources for drug development and the need for expedited drug approval, drug developers have turned, in part, to the strategy of repurposing agents for which safety and clinical data are already available. Approval of drugs that are already in clinical use for one indication and are being repurposed for another indication is inherently faster and more cost effective than for new agents that lack regulatory approval of any sort. There are four known growth factors which have been repurposed in the recent past as radiomitigators following the FDA Animal Rule: Neupogen, Neulasta, Leukine, and Nplate. These four drugs were in clinic for several decades for other indications and were repurposed. A large number of additional agents approved by various regulatory authorities for given indications are currently under investigation for dual use for acute radiation syndrome or for delayed pathological effects of acute radiation exposure. The process of drug repurposing, however, is not without its own set of challenges and limitations.

## Introduction

Exposure to intense ionizing radiation will evoke invariably significant damage to selective tissues of vital organ systems of the body; most notably, the vascular, blood-forming, gastrointestinal, and reproductive systems. Following such intense irradiation, acute lymphohematopoietic tissue damage rapidly manifests as evidenced by rapid changes in clinically relevant blood parameters, namely by fast, time-dependent decreases in blood cell concentrations (specifically lymphocytes, granulocytes, and thrombocytes/platelets) (Gale et al., 2019).

The degree and frequency to which these major ionizing radiation-induced pathophysiological responses are expressed are clearly dependent on a multitude of factors that encompass not only the nature of the radiation exposure, but also the extent and location of bodily exposure. These response variables are superimposed on the all-important biological makeup of the exposed individual (age, gender, overall health status, as well as basic physiologic and genetic constitutions of the exposed individual). Lest we forget that ‘time’ is key in order to bring the physics of radiation together with biological elements in order for these irradiation pathologies to fully develop and be expressed.

At acute radiation doses of >10 Gy with high dose rates, exposed individuals will die very quickly (i.e., hours to a few days) from largely untreatable neurovascular effects. At doses of >2 to <10 Gy, injury to hematopoietic tissue (bone marrow) and the gastrointestinal (GI) tract is obvious (Hall and Giaccia, 2012). Survivors of such radiation exposures will also fall subject to the delayed effects (known as delayed effects of acute radiation exposure, DEARE), which may include involvement of other organs such as the lungs, kidneys, and heart (Singh and Seed, 2020b). These sub-syndromes, their animal models, mechanism of injury, and medical countermeasures have been discussed in several reviews (Williams et al., 2010a; Williams et al., 2010b; Williams and McBride, 2011; Williams et al., 2012; Williams J. P. et al., 2016). Despite substantial efforts over the last several decades to advance effective and safe radiation medical countermeasures for acute radiation syndrome (ARS) and for DEARE, a limited number of countermeasures have been fully approved by the Food and Drug Administration (FDA) for clinical use for humans (Singh et al., 2017a; Singh et al., 2017b; Singh and Seed, 2017; Singh and Seed, 2020b). Thus, there is a serious need for government agencies, academicians, and corporate entities to make a joint effort to get multiple agents that can be used either before or after irradiation approved for each indication in the shortest possible time. There are several promising radiation countermeasures under investigation for regulatory approval for ARS and DEARE (Singh et al., 2017a; Singh et al., 2017b; Singh and Seed, 2017; Singh and Seed, 2020a).

Under any mass casualty event due to a nuclear/radiological scenario, the number of individuals requiring medical care will be enormous, but the real number of people critically injured by high doses of radiation will be limited (Singh and Seed, 2020b). The radiation-injuries may be grouped based on ‘time’ to manifest radiation injury and etiology of the injuries. Based on the ‘time,’ injuries may be grouped in terms of early/acute, delayed, or late occurrence. Further, they can be characterized as being of either ‘deterministic’ or ‘stochastic’ origins, with the ‘early arizing’ pathologies dominated by ‘deterministic’ responses, while the ‘late-arizing’ responses divided more evenly by the prevalence of long-developing pathologies of a ‘stochastic’ nature. It should be noted that the ‘deterministic’ responses/pathologies often share common features regardless of whether they arise relatively early following acute exposures or if they arise relatively late following various exposure modalities (e.g., acute, chronic, fractionated exposures). By contrast, a sizable fraction of the late-arizing pathologies have a stochastic, probabilistic nature, with ‘cancer’ being the group’s arch type (Seed et al., 1984; Seed et al., 1985; Upton, 1985; Singh and Seed, 2020b).

Cancer that develops long after radiation exposure is often associated both temporally and causally with initial mutagenic processes. A commonly held concept, with ample experimental support, is that cancer risks following radiation exposures (or exposures of other types of toxic physiochemical agents) can be substantially mitigated pharmacologically by blocking exposure-related mutagenesis (Grdina et al., 1985; Grdina et al., 2000; Grdina et al., 2002; Singh and Seed, 2019). In this regard, there are numerous categories of anti-cancer drugs that are currently in use with some having anti-mutagenic activity as well. These drugs include alkylating agents, anti-metabolites, natural products, and hormones (Ali et al., 2012). The more prominent of these agents specifically designed to limit toxicant-induced mutagenesis include phosphorothioates (amifostine/WR1065) and nitroxides (Tempol) (Johnstone et al., 1995; Grdina et al., 2000; Grdina et al., 2002; Seed et al., 2002). Clearly, there is a significant opportunity for drug developers and the pharmaceutical industry to repurpose previously FDA-approved drugs for the treatment of various types of cancers, particularly to confer radiation-enhancing effects. Some promising agents include aspirin, statins, and metformin which have the ability to enhance outcomes in cancer patients by decreasing the radiation dose, and also reduce the treatment expense (Khan et al., 2019). The reader should note, however, that we have limited the scope of this article to only acute and delayed effects of irradiation and not to ‘cancer,’ per se.

The targeting specificity of drugs in clinical use for given indications appears to decrease over time as new information accumulates (Papapetropoulos and Szabo, 2018). Identification of additional molecular targets for a drug may be an issue for the therapeutic agent already under clinical use from a specificity perspective. At the same time, such additional information regarding new targets and associated activities for a given drug may offer further therapeutic potential, leading to the repurposing of the same drug for another indication. Drug repurposing (also known as dual use) is another way of utilizing known drugs for other indications. The specific drug might be in the clinic for a specific indication, or may be withdrawn from development as a result of issues related to efficacy, side effects, or commercial considerations. Drug rescuing is a similar phenomenon; it is the process(es) that refers to circumstances where the failed agent for one indication is investigated later with the objective of introducing it for another indication (Sharlow, 2016). Such repurposing efforts in the drug development arena are vital to having enough drugs for various indications with limited resources.

A current, prime example of the latter ‘repurposing process’ involves the antiviral agent, remdesivir (Gilead Sciences, Inc., Foster City, CA) and its use in treating severely ill COVID-19 infected patients (Gilead Sciences Inc., 2020). This agent was originally part of a bank of antivirals set aside by Gilead that was deemed in initial clinical testing to be ineffective in managing SARS (severe acute respiratory syndrome)-like infections, but was later rescued, clinically retested, and subsequently brought through the FDA’s regulatory approval process in record time in order to pursue it as an effective remedy for the current raging COVID-19 (Coronavirus disease 2019) pandemic (US Food and Drug Administration, 2020b).

Conventional drug development has a roughly 90% attrition rate; this is to say that 90% of the candidate drugs studied in preclinical models that lack toxicity in small as well as large animals and that are well accepted by human studies, ultimately never receive regulatory approval for clinical use (Mullard, 2016). Second attempts to redevelop drugs already in clinical evaluation saves time and money. The time saved has been estimated to be in the range of 12–14 years, and the overall cost saved for FDA approval is in the range of $40–80 million. The latter compares to the >$2 billion to develop an agent from the initial *in vitro* work and associated ‘hit selection’. The number of new drugs approved by regulatory agencies per billion USD spent for development has been reduced to one half every 9 years since 1950, underscoring the declining efficiency of drug development (Kakkar et al., 2018).

There is also a distinct possibility of failure in this repurposing route as well; a possibility that also increases the overall cost for successful repurposing (Ishida et al., 2016; Cha et al., 2018; Gelosa et al., 2020). There is another fact which needs to be taken into consideration in favor of repurposing. A significant proportion of funding for such repurposing goes to the large Phase III trials that are mandatory in order to validate the efficacy for the repurposed drug. The high cost associated with such Phase III trials is due to the large numbers of patients that are generally needed for regulatory approval. Furthermore, the repurposed medicinals may not require an approval for use in patients. If the repurposed drug demonstrates robust efficacy for a second indication, medical professionals may prescribe such drugs off-label, specifically for diseases which have limited treatment options.

Drug development programs for medical countermeasures designed for radiation-induced ARS and related radiation-injuries are restricted in a regulatory sense, as they are being developed using the FDA Animal Rule and cannot be evaluated for efficacy in a clinical setting due to ethical reasons (Allio, 2016; U.S. Food and Drug Administration, 2015a).

### FDA Approved Agents Repurposed for ARS

Four growth factors/cytokines approved by the US FDA for several indications were in clinic for several decades. These agents were repurposed as radiomitigators for ARS, or more specifically for H-ARS (a hematopoietic sub-syndrome of ARS), following the Animal Rule during the last six years (U.S. Food and Drug Administration, 2015a). These agents are Neupogen (filgrastim), Neulasta (PEGylated filgrastim), Leukine (sargramostim), and Nplate (romiplostim) (Table 1) (Amgen Inc., 2015a; Amgen Inc., 2015b; Farese and MacVittie, 2015; National Institute of Allergic and Infectious Diseases, 2015; U.S. Food and Drug Administration, 2015b; U.S. Food and drug Administration, 2018b; Sanofi-Aventis U.S. LLC, 2018; Singh and Seed, 2018; Clayton et al., 2020; Wong et al., 2020a; Wong et al., 2020b; Singh and Seed, 2020b; Zhong et al., 2020; Amgen Inc., 2021; Gale and Armitage, 2021). The data for these growth factors in context of their human use as radiation countermeasures have been recently reviewed (Farese and MacVittie, 2015; Singh and Seed, 2018; Singh and Seed, 2020b; Wong et al., 2020a; Wong et al., 2020b; Zhong et al., 2020; Gale and Armitage, 2021). These articles also discuss various types of medical management used for testing these agents in large animal model.

**TABLE 1:** US FDA-approved growth factors for other indications repurposed for H-ARS as radiomitigators.

FDA-approved drugs	Indications of earlier approval	Repurposed indication	Date of repurpose	Comments	References*
Neupogen/filgrastim/G-CSF	Five different indications of neutropenia and for mobilization of autologous hematopoietic progenitors	Adult patients of H-ARS	March 2015	Requires full supportive care: blood products and individualized antibiotics; can be delayed up until 24 h after irradiation	Farese and MacVittie (2015); U.S. Food and Drug Administration (2015); Amgen Inc. (2015)
Neulasta/PEGylated filgrastim/PEGylated G-CSF	Decreases infections as displayed by febrile neutropenia	Adult and pediatric patients of H-ARS	November 2015	Needs full supportive care: blood products and personalized antibiotics; can be delayed until one day after irradiation	National Institute of Allergic and Infectious Diseases (2015); Amgen Inc. (2015)
Leukine/sargramostim/GM-CSF	Five different indications for shortening time to neutrophil recovery, mobilization of hematopoietic progenitors, acceleration of myeloid reconstitution, and treatment of delayed neutrophil recovery	Adult and pediatric patients of H-ARS	March 2018	Requires minimal supportive care: antibiotics and fluid; can be delayed until 48 h after irradiation	U.S. Food and drug Administration (2018); Singh and Seed (2018); Sanofi-Aventis U.S. LLC (2018); Gale and Armitage (2021); Zhong et al. (2020)
Nplate/romiplostim/synthetic TPO	Adult and pediatric patients of immune thrombocytopenia	Adult and pediatric patients of H-ARS	January 2021	Requires minimal supportive care: antibiotics and fluid; should be administered as soon as possible after suspected or confirmed exposure to radiation	Amgen Inc. (2021); Wong et al. (2020); Wong et al. (2020)

The NHP studies were performed at different research sites under the control of respective IACUC rules that may well have dictated that studies of lethal effects must include blood transfusions. The specific medical countermeasures was tested with and without blood transfusions as required based on a trigger-to-treat.

Filgrastim, PEGylated filgrastim, and sargramostim have some basic structural differences. Sargramostim is a glycosylated product prepared in an expression system using *Saccharomyces cerevisiae,* while filgrastim is a product of the *Escherichia coli* expression system and is not glycosylated. Furthermore, the comparison of efficacy and treatment outcomes of these two countermeasures is not relevant since these two proteins bind to different receptors (Gale and Armitage, 2021). Receptors for filgrastim/G-CSF (granulocyte colony-stimulating factor) and sargramostim/GM-CSF (granulocyte-macrophage colony-stimulating factor) belong to the well-known cytokine receptor family. Differences in the expression of receptors are responsible for the functional disparities between filgrastim and sargramostim (Gale and Armitage, 2021). Biological activity may also depend on how sargramostim is processed. Such distinctions result in differences in the efficacy and safety profiles of these two agents in clinical settings (Stull et al., 2005). Filgrastim use is significantly greater than sargramostim in most hematology and oncology settings.

Data gathered from preclinical testing using non-human primates (NHPs) suggest differences in optimal time of drug administration after radiation exposure and the intensity of supportive care required for the above four agents. The results of these NHP studies have been reviewed thoroughly relative to the various testing conditions employed with these four countermeasures (Wong et al., 2020a; Wong et al., 2020b; Gale and Armitage, 2021). The optimal time for filgrastim and PEGylated filgrastim treatment initiation is 24 h post-irradiation, as opposed to 48 h post-irradiation for sargramostim. Filgrastim/PEGylated filgrastim is effective with full supportive care including blood products and individualized antibiotics. Filgrastim was not effective with minimal or no supportive care (Gluzman-Poltorak et al., 2014; Farese and MacVittie, 2015). Sargramostim and romiplostim were found to be effective with moderate supportive care without blood products. It has not been investigated with intensive supportive care/blood products or without any supportive care (Wong et al., 2020a; Wong et al., 2020b; Gale and Armitage, 2021). Sargramostim is available in lyophilized form, which may offer benefits under situations of limited infrastructure. PEGylated filgrastim has an advantage over the other two when availability of medical personnel is limited, as only two doses are needed. It is important to note that direct comparisons of these four agents in respect of time of administration and supportive care in NHP models are lacking and need additional investigations. Additional drawbacks of these growth factors is their cost and storage conditions (specifically for Neupogen and Neulasta). Furthermore, these agents lack radioprotective efficacy when administered prior to irradiation and have limited potential for long-term storage.

There is significant knowledge for the use of these agents after radiological or nuclear (Rad/Nuc) accidents in humans that, in general, support the concept that they serve to accelerate bone marrow recovery and improve survival-based outcomes (Singh et al., 2015). Unfortunately, due to the observational nature of such studies, ‘essential control’ subjects are missing and claims of efficacy and safety in humans exposed to Rad/Nuc accidents remain, accordingly, untested. Nevertheless, these agents were approved following the Animal Rule of the FDA and three agents are available in the US Strategic National Stockpile/Vendor Managed Inventory.

### Regulatory Agency Approved Drugs for Repurposing for the Treatment of Radiation Injury

There are several agents approved for human use for a large number of indications by regulatory agencies which are being investigated in various laboratories to repurpose for ARS, DEARE, and other types of late-arizing injuries that arise from either non-acute external exposures or from internally deposited radionuclides.

### Agents Being Investigated for Repurposing for ARS

In addition to the four FDA-approved radiomitigators mentioned above, several previously FDA-approved agents are being evaluated for possible expanded indications for preventing, mitigating, or treating accidental or unwanted radiation injuries (Table 2). A selected number of these prospectively useful agents are listed below and are briefly described. We have focused on agents which have FDA approval for one or more indications and are being investigated as radiation countermeasures either alone or in combination with another agent.

**TABLE 2:** US FDA-approved drugs for other indications currently being investigated for repurposing for acute and delayed effects of radiation exposure.

FDA-approved drugs	Indication(s) for FDA approval	Indication for repurposing	Comments	Relevant references*
Amifostine/Ethyol	Reduces renal toxicity as a result of cisplatin treatment for ovarian cancer, reduces irradiation-induced xerostomia in head and neck cancer patients	H-ARS	Promising agent but dose required for protection from ARS has severe side effects/toxicity	Singh and Seed (2019); Cumberland Pharmaceuticals Inc. (2017)
Promacta/Doptelet	Thrombocytopenia/idiopathic thrombocytopenic purpura (ITP)	H-ARS	These agents stimulate platelet/thrombocyte production and hematopoietic recovery	Parameswaran et al. (2014); Jacobson et al. (2017); Erickson-Miller et al. (2009); U.S. Food and Drug Administration (2018); Dova Pharmaceuticals (2019); Shirley (2018)
Capoten/Vasotec/Prinivil/Ramipril	Angiotensin-converting enzyme inhibitors, indicated for hypertension	Radiation injury of lung and kidney/DEARE	Helpful for early and late-arizing radiation injuries as a prophylaxis and mitigator	Singh et al. (2017); U.S. Food and Drug Adsministration (2015); Rosen et al. (2015)
Palifermin	Indicated for oral mucositis in individuals with hematopoietic stem cell transplantation for malignancies	ARS	Truncated N-terminal recombinant keratinocyte growth factor	Vadhan-Raj et al. (2013); Lauritano et al. (2014); Johnke et al. (2014); Rosen et al. (2015)
Erythropoietin	Indicated for anemia linked to renal dysfunction	ARS	Used during the accidents of Tokai-mura, Japan and Henan Province, China	Gianoncelli et al. (2015); Agarwal and McBride (2016); Covic and Abraham (2015); Nagayama et al. (2002); Liu et al. (2008)
IL-3	Not fully approved by the FDA for ARS	ARS	Used during the accident of Nyasvizh, Belarus and Soreq, Israel	Nabholtz et al. (2002); International Atomic Energy Agency (1993); International Atomic Energy Agency (1996)
IL-11/Oprelvekin	Indicated for thrombocytopenia after myelosuppressive chemotherapy in individuals with non-myeloid malignancies	ARS	Radiomitigator and radioprotector	No author listed (1998); Potten 1995; Potten (1996); Burnett et al. (2013); Hauer-Jensen (2014)
Statin	Indicated for atherosclerosis and hypercholesterolemia	ARS	Anti-inflammatory due to inhibition of 3-hydroxy-3-methylglutaryl coenzyme A reductase	Gotto (2003); Grobbee and Bots (2003); Diomede et al. (2001); Sizar et al. (2020); Williams et al. (2004); Wang et al. (2007)
Pentoxifylline	Indicated for claudication and pain in case of occlusive peripheral arterial disease	H-ARS	Methyl xanthine derivative with immunomodulating, anti-inflammatory, antioxidant, and vascular effects	McCarty et al. (2016); Boerma et al. (2008); Berbée et al. (2011); Kulkarni et al. (2013)
Xigris	Indicated for high risk sepsis linked with acute organ dysfunction	ARS	Recombinant human activated protein C	Aneja and Fink (2007); Bernard et al. (2001); Griffin et al. (2006); Geiger et al. (2012)
CpG-ODN	Approved as adjuvants for vaccines	ARS	Stimulates innate as well as adaptive immune responses	Fehér (2019); Zhang et al. (2013); Zhang et al. (2011); Zhang et al. (2011)
Auranofin/Ridaura	Indicated for rheumatoid arthritis	ARS	It is an anti-inflammatory, anti-cancer, neuroprotective, and cardioprotective agent	Nag et al. (2019); Nardon et al. (2016)
Diclofenac sodium	Indicated in osteoarthritis, rheumatoid arthritis, and ankylosing spondylitis	ARS	Benzene-acetic acid derivative	US Food and Drug Administration (2020); Novartis (2005); Alok et al. (2013)
Metformin	Indicated for type 2 diabetes	ARS	Used for FDA-approved as well as for off-label indications	Corcoran and Jacobs (2020); Miller et al. (2014); Xu et al. (2015)
Surfaxin	Indicated for the prevention of respiratory distress syndrome in infants	Lung injury/DEARE	Helpful for mitigation of lung injury after targeted thoracic irradiation	Piehl and Fernandez-Bustamante (2012); Christofidou-Solomidou et al. (2017)
Diethylcarbamazine citrate	An anti-filarial drug used to treat filariasis	Lung injury/DEARE	Known for anti-fibrotic, anti-oxidative, anti-inflammatory, and anti-carcinogenic properties	Hawking (1979); Queto et al. (2010); Farzipour et al. (2020)
Mozobil	Indicated for autologous transplantation in cases of non-Hodgkin’s lymphoma and multiple myeloma	H-ARS	With G-CSF and agents inducing G-CSF increases peripheral blood CD34^+^ cells, increases neutrophils	De Clercq (2019); De Clercq (2009); De Clercq (2010); Singh et al. (2014); Singh et al. (2014); Singh et al. (2010); Singh et al. (2012); Singh et al. (2013); Dykstra (2017)
Silverlon	Indicated for blister injuries caused by sulfur mustard, wound and burn contact dressing	Cutaneous radiation injuries	Received FDA approval for multiple indications since 2003	DiCarlo et al. (2018); Argentum Medical (2019); Aurora et al. (2018); Pozza et al. (2014)
Ciprofloxacin	Indicated for severe infections caused by Gram-negative bacteria	ARS and combined injury	Effective in murine model of irradiation and wound	Imrie et al. (1995); Fukumoto et al. (2014); Fukumoto et al. (2013); Sahakitrungruang et al. (2011)

#### Amifostine

As mentioned above, only limited drugs have been approved by the US FDA to counter radiation injury, although several additional agents are currently under investigation. The thiol group in amifostine {WR-2721, 2-[(3-aminopropyl)amino]ethanethiol dihydrogen phosphate, Ethyol (trihydrate form of amifostine)} acts as a free radical scavenger, and this group of compounds represents one of the most effective classes of radioprotectors (Grdina et al., 2000; Kouvaris et al., 2007). However, they are generally not well tolerated due to a number of side effects that serve to limit the optimal dosing required for radioprotection of ARS (Singh and Seed, 2019). Preclinical studies using animal models suggested that amifostine protects normal tissues from injuries caused by irradiation (Rasey et al., 1988; Singh and Seed, 2019). Common side effects of such agents include vomiting, diarrhea, and hypotension. These effects result in adverse behavioral responses and decreased performance (Bogo et al., 1985). The FDA has approved amifostine for limited clinical use (Singh and Seed, 2019). In brief, amifostine, however promising as a radioprotector, has not been considered suitable for use in the general civilian population, high-risk individuals, or special operation military forces.

Amifostine is fully approved by the US FDA for these two clinical indications: 1) to diminish renal toxicity associated with the use of chemotherapy of cisplatin in patients with ovarian cancer, and 2) to decrease xerostomia in individuals undergoing post-operative radiotherapy for head and neck cancers (Brizel et al., 2000; Cumberland Pharmaceuticals Inc., 2017). The enhanced absorption of amifostine by the kidney and salivary glands may be a significant contributor for the noted protection of these relevant organs and tissues.

Significant attempts have been made by scientists, academicians, and government agencies to render amifostine more useful by reducing its side effects and toxicity by developing a large number of novel analogs, new formulations, and also new drug delivery approaches. The objective of such efforts is to preserve or improve the efficacy and also prolong the therapeutic window. Specifically, amifostine is being extensively investigated in various laboratories to make it more user-friendly (Seed, 2005; Seed et al., 2014). Such efforts include: 1) the synthesis of better tolerated analogs with minimal side effects (Davidson et al., 1980; Brown et al., 1988), 2) the identification of additional molecular targets using omics approaches (Cheema et al., 2019; Seed et al., 2019; Singh and Seed, 2019; Cheema et al., 2020), 3) the development of synergy to combine lower doses of amifostine and other effective pharmacologicals with limited or no side effects (Srinivasan et al., 1992; Singh et al., 2016), 4) the use of another agent to reduce its emetic effects (Seed, 2005), and 5) the optimization of the efforts for ‘slow-release’ delivery (Fatome et al., 1987; Srinivasan et al., 2002; Pamujula et al., 2010). These approaches proved useful to a limited extent in reducing its side effects and toxicity, but have not been successful in eliminating its toxicity completely. Recently, these approaches to make amifostine more useful have been reviewed (Singh and Seed, 2019). We believe that amifostine, with its several positive characteristics, deserves more investigation in order to pursue and eventually receive full regulatory approval for generalized use outside clinical settings for radiation exposure emergencies.

The poly-pharmacy approach appears to be very encouraging, where low doses of amifostine are being combined with other radiation countermeasures under development with the objective to increase the efficacy of amifostine and avoid its side effects. There are several agents that have been tested in combination with amifostine, and results of some of these studies are encouraging (Singh et al., 2016; Singh and Seed, 2019). Specifically, amifostine has been tested in combination with growth factors/cytokines (G-CSF), metformin, antioxidative agents (other thiols), vitamin E components (tocopherol and tocotrienol), prostaglandin E2, β-glucan, Broncho-Vaxom, and a polysaccharide from *Sipunculus nudus*, etc [recently reviewed (Singh and Seed, 2019)].

#### Mozobil

Mozobil (AMD3100 or plerixafor) was initially identified as an anti-HIV agent (De Clercq, 2019). It specifically blocks the CXCR4 receptor, the co-receptor of T-lymphotropic HIV strains. Mozobil mobilizes the CD34^+^ hematopoietic progenitors to blood by blocking the chemokine receptor (CXCR4) and disturbing CXCR12 and CXCR4 interaction, which is responsible for tethering stem cells to bone marrow cells (Broxmeyer et al., 2005). Such CD34^+^ cell mobilization occurs when mozobil is used either alone or with G-CSF. Mozobil received US FDA approval for transplantation in individuals with multiple myeloma or patients with Non-Hodgkin’s Lymphoma (NHL) in 2008 (De Clercq, 2019). It can also be used in other malignancies and hereditary disorders (e.g., hepatopulmonary syndrome and WHIM, a congenital immune deficiency). New antagonists of CXCR4, KRH 1636, and CX0714 have also been reported that are not structurally similar to mozobil but may behave in a similar fashion (De Clercq, 2009; De Clercq, 2010).

There are several reports where mozobil has been used in combination with radiation countermeasures inducing G-CSF (e.g. γ-tocotrienol, tocopherol succinate) in animal models to mobilize progenitors, and such mobilized progenitors have been successfully used to protect mice exposed to supralethal doses of radiation which lead to GI-ARS and H-ARS (Singh et al., 2010; Singh et al., 2012; Singh et al., 2013; Singh et al., 2014a; Singh et al., 2014b). These mobilized progenitors have been administered to mice several days after radiation exposure and still retain significant survival benefits. It has been discussed that mozobil along with G-CSF increases blood CD34^+^ cells that may result in improved neutrophils. Such combination also increases other blood cells leading to better outcomes in patients receiving irradiation (Dykstra, 2017).

### Cytokines, Pro-Inflammatory Cytokine Inhibitors and Related Chimeric Recombinants

Several cytokines mitigate radiation injury in tissues and also accelerate tissue recovery. As noted earlier, four growth factors/cytokines have already been repurposed as radiomitigators for ARS, specifically H-ARS. Other type-specific or general classes of cytokines/growth factors need to be reevaluated as potentially useful countermeasures for acute radiation exposures. For example, recombinants that selectively inhibit cytokines (pro-inflammatory) also reduce fibrosis and late tissue injury linked to irradiation, or chimeric growth factors such as myelopoietin. An efficacious (as shown in preclinical animal models) chimeric recombinant, does the same in terms of mitigating acute hematopoietic injury. Some cytokines have been approved by the FDA for other indications, and efforts are being made to develop these agents as radiation countermeasures.

#### Palifermin

This is the truncated N-terminal keratinocyte growth factor, and this agent is also called as fibroblast growth factor-7. It stimulates epithelial cell proliferation and acts in a paracrine manner. It has its effects on various tissues such as the hepatocytes of the liver, intestine, type II pneumocytes of the lung, keratinocytes in squamous epithelia, hair follicular cells, and transitional urothelial cells (Danilenko, 1999; Farrell et al., 1999). Palifermin received FDA approval to prevent oral mucositis in individuals undergoing stem cell transplant for hematological cancers (Vadhan-Raj et al., 2013; Johnke et al., 2014; Lauritano et al., 2014). This agent is useful for repair and protection of epithelial cells through fibroblast growth factor receptor-2b (FGFR-2b). Its efficacy is a result of stimulation of cell proliferation and being anti-apoptotic (Finch et al., 2013). Palifermin treatment has been shown to prevent radiation-induced gastrointestinal injury in mice (Cai et al., 2013). Palifermin has also been shown to ameliorate radiotherapy and chemotherapy-induced mucosal toxicity (Finch et al., 2013).

#### Erythropoietin

There are several bioengineered analogs of EPO such as Aransep, Epoetin, Epogen, Darbepoetin, and Procrit. These agents are approved by the FDA for various hematologic indications. Their primary indication is for the treatment of severe anemia via stimulation of erythropoiesis (associated with chronic renal dysfunction) as a result of intense chemotherapy or radiotherapy (Covic and Abraham, 2015; Gianoncelli et al., 2015; Agarwal and McBride, 2016). These recombinant agents are not approved by the U.S. FDA as a radiation medical countermeasure for use in radiation casualty scenarios. Regardless of this current regulatory status, it is commonly believed that these agents would be used by attending physicians if clinical conditions of radiation exposed patients called for their use. In this regard, it is important to note that EPO was indeed used during the radiological accidents of Tokai-mura in Japan and Henan Province in China for the treatment of exposed victims (Nagayama et al., 2002; Liu et al., 2008).

#### Interleukin-3

Although recombinant IL-3 stimulates myelopoiesis, this agent has not been pursued actively as a medical countermeasure for ARS or for chemotherapy-induced myelosuppression (Hammond et al., 1990; Lord et al., 1991). Furthermore, leridistim (myelopoietin, a chimeric dual G-CSF and IL-3 receptor agonist) is not being developed as a countermeasure, despite promising initial results showing its efficacy in ameliorating severe, radiation-induced neutropenia within large, experimental animals (MacVittie et al., 2000). A phase III trial with leridistim and G-CSF demonstrated G-CSF to be better than leridistim in preventing chemotherapy-induced neutropenia (Nabholtz et al., 2002). Although IL-3 is not approved by the U.S. FDA as a radiation medical countermeasure, it has been used for treating radiation-exposed victims of Israel–Soreq and Belarus–Nyasvizh with positive treatment outcomes (International Atomic Energy Agency, 1993; International Atomic Energy Agency, 1996) and accordingly, there is a basis for further investigating this agent as a radiation medical countermeasure.

#### Interleukin-11 (IL-11, Oprelvekin)

Oprelvekin is recombinant IL-11 and is produced in *E. coli*. It has a molecular weight of 19 kDa and is non-glycosylated due to being a product of *E. coli*. This polypeptide is 177 AA compared with 178 AA in the natural IL-11. Oprelvekin binds to the IL-11R leading to a signal transduction cascade. In preclinical models, Oprelvekin (Neumega) demonstrated potent thrombopoietic activity during compromised hematopoiesis. It is an FDA approved drug and is indicated for thrombocytopenia in individuals with non-myeloid malignancies following myelosuppressive chemotherapy (No author listed, 1998). It is also used for the therapy of inflammatory bowel disease. IL-11 has been used for acute and chronic radiation injuries as part of cytokine treatment (Seed et al., 2001). In a murine model of radiation injury, IL-11 has been shown to improve crypt counts and reduce mucosal injury (Potten, 1995; Potten, 1996). This agent has been used before as well as after irradiation, suggesting that it is both a radioprotector and a radiomitigator (Potten, 1995; Burnett et al., 2013), but its route of administration needs investigation to avoid significant toxicity (Hauer-Jensen, 2014).

#### Thrombopoietin

Treatment for thrombocytopenia as a result of high dose radiation exposure is an important area of radiation countermeasure development, as severe thrombocytopenia can be life-threatening and is a good indicator of mortality risk (Stickney et al., 2007). The thrombocytopenia was a better predictor of mortality of acutely irradiated rhesus NHPs when compared to comparatively measured parameters of neutropenia. There is significant discussion about the possibility that TPO or a related growth and development factor can exert significant survival benefit as demonstrated in preclinical irradiated animal models. Specifically, TPO increases survival benefits in irradiated mice. It has also been shown to increase hematopoietic recovery in the C57BL/6J strain of mice at a dose of 0.3 µg/mouse when administered either 2 h before or 2 h post-irradiation (Mouthon et al., 1999; Mouthon et al., 2002). Finally, in acutely radiation exposed victims of the Tokai-mura criticality accident, TPO has been reported to be beneficial (Nagayama et al., 2002).

Several molecules targeting TPO receptor binding sites have been developed. Nplate (also known as romiplostim) and Promacta of Amgen and Glaxo SmithKline, respectively, have been approved by the FDA to treat idiopathic thrombocytopenic purpura (Kuter, 2007). Romiplostim is a synthetic TPO agonist and is FDA-approved for the treatment of low platelet counts in individuals with immune thrombocytopenia. It binds to TPO c-Mpl receptor, preferentially stimulates platelet generation in the bone marrow (Parameswaran et al., 2014; Jacobson et al., 2017), and also possesses a peptide domain recognized by the TPO receptor linked to an Fc carrier domain that increases its plasma half-life. The pharmacodynamics of romiplostim and its effect on platelet production in rodents and NHPs (rhesus and cynomolgus) have been investigated with both intravenous (*iv*) bolus and subcutaneous (*sc*) administrations (Krzyzanski et al., 2013).

Romiplostim has been shown to protect γ-irradiated C57BL/6J mice when administered intraperitoneally (*ip*) 2 h post-irradiation and continued for 3 or 5 days as a daily administration at a dose of 50 μg/kg (Yamaguchi et al., 2018). At 30 days post-irradiation, complete blood cell counts of irradiated and romiplostim-treated mice were comparable to unirradiated mice. It was not effective, however, if treatment was started 24 h or 48 h post-irradiation (9 Gy), or if higher doses of radiation (>9 Gy) were used (Mouthon et al., 2002). In another study, this agent was found to increase mouse survival, support hematopoiesis, reduce injury to tissues, increase mesenchymal stem cells in the spleen, and suppress several miRNA linked to radiation-induced leukemogenesis (Yamaguchi et al., 2019). Furthermore, ∼40% survival enhancement was observed after administration of this agent as a single dose of 30 μg/kg. Its combination with PEGylated filgrastim or more frequent dosing did not show any additional benefit (Bunin et al., 2020). In a recent study, several proteins (out of 269 proteins) were found to be increased in the serum of C57BL/6J mice (keratin, type II cytoskeletal 1, fructose-1, 6-bisphosphatase, cytosolic 10-formyltetrahydrofolate dehydrogenase, peptidyl-prolyl *cis*-trans isomerase A, glycine N-methyltransferase, glutathione S-transferase Mu 1, regucalcin, fructose-bisphosphate aldolase B and betain–homocysteine S-methyltransferase 1) exposed to a lethal dose of ^137^Cs γ-rays whole-body radiation. Treatment with romiplostim administered *ip* daily at a dose of 50 μg/kg for 3 days starting 2 h post-irradiation decreased these proteins. These proteins were suggested to be indicators of the high-dose radiation injury (Nishida et al., 2020).

Romiplostim and PEGfilgrastim administration, either alone or in combination, provided improvements in hematology compared with control NHPs without any treatment (Wong et al., 2020b). The combination of both drugs demonstrated the most significant hematological (particularly neutrophils and platelets) benefits. The above results supported the development of romiplostim as radiation medical countermeasures for use in humans. This agent has been used off-label in a phase II clinical study for efficacy and safety for thrombocytopenia in chemotherapy patients (Wang et al., 2012). There were a few treatment-associated adverse events resulting from romiplostim use, including cerebrovascular accidents and increases in bone marrow blasts.

In another study, different combinations of erythropoietin (EPO), G-CSF, romiplostim, and nandrolone decanoate were administered to female C57BL/6J Jcl mice 2 h post-irradiation with 7 Gy (100% lethal). The administration of romiplostim in combination with EPO and G-CSF for 5 days starting soon after radiation exposure provided 100% protection in mice when there was 100% mortality in untreated control animals. The CBC analyses demonstrated complete hematologic recovery in irradiated and drug-treated surviving animals (Hirouchi et al., 2015).

Recently, romiplostim was evaluated in male and females NHPs without blood products. Administration of romiplostim following irradiation of NHPs resulted in improved survival and hematological profiles (Wong et al., 2020a; Wong et al., 2020b). In the latest study, there were 20 males and 20 females in each treatment and vehicle group (Wong et al., 2020a). Further improvements were noted when romiplostim was used in combination with PEGfilgrastim. Survival was better in males compared with females. In January 2021, romiplostim received FDA approval for the treatment of radiation exposed adult and pediatrics patients (Amgen Inc., 2021). The recommended dose of Nplate is 10 μg/kg once as a *sc* injection as soon as possible after exposure to more than 2 Gy.

Eltrombopag (also known as Promacta) is another FDA-approved agonist of TPO receptor indicated for immune thrombocytopenia, chronic hepatitis C patient thrombocytopenia, and patients of aplastic anemia. This agent has a long half-life and is reported to be orally effective. It boosts megakaryocytes and promotes a rise in platelet counts. It has demonstrated a significant increase in platelet counts in chimpanzees when administered at 10 mg/kg/day for 5 days (Erickson-Miller et al., 2009). The development path for this agent is difficult since it is found to be effective only in chimpanzees and humans. It is not effective in other animal models. Due to the lack of access of the full array of animal models that might be applied in preclinical evaluations of the drug as a potential radiation countermeasure, its development has limitations. The species specificity of eltrombopag is due to the presence of a histidine (499 of human TpoR), which is required for binding (Erickson-Miller et al., 2009; DiCarlo et al., 2011a). There is a lysine residue in place of histidine in all other animals investigated (DiCarlo et al., 2011b). It is not easy to use chimpanzees for the development of radiation medical countermeasures or for the development of any drug due to its endangered status. Romiplostim and eltrombopag are also investigated as a treatment for thrombocytopenia of other etiologies (DiCarlo et al., 2011a; DiCarlo et al., 2011b; Liesveld et al., 2013).

Avatrombopag (Doptelet; Dova Pharmaceuticals) is used for the treatment of thrombocytopenia. This is a second-generation small molecule thrombopoietin receptor agonist that is orally bioavailable. It activates thrombopoietin receptors and increases megakaryocyte proliferation/differentiation and platelet production (DiCarlo et al., 2018; Shirley, 2018; U.S. Food and Drug Administration, 2018b; Dova Pharmaceuticals, 2019). Avatrombopag has received its FDA approval for the treatment of periprocedural thrombocytopenia with chronic liver disease. Thrombocytopenia potentially affects the management of chronic liver disease due to the increased risk of bleeding during surgery or liver biopsy. It is also recommended for immune thrombocytopenia and as an alternative to platelet transfusions (Cheloff and Al-Samkari, 2019; Dlugosz-Danecka et al., 2019; Xu and Cai, 2019; Poordad et al., 2020). Lusutrombopag (Mulpleta) is another FDA-approved thrombopoietin receptor agonist which is orally effective for thrombocytopenia in patients with chronic liver disease (Abdela, 2019; Clemons Bankston and Al-Horani, 2019). There are obvious reasons to attempt to develop these agents as pharmacologic countermeasures to acute radiation injuries (DiCarlo et al., 2018; Shirley, 2018; U.S. Food and Drug Administration, 2018a; Dova Pharmaceuticals, 2019).

ALXN4100TPO is another TPO receptor agonist that has the very useful clinical attribute of reducing the potential for the generation of endogenous TPO antibodies. It was shown to increase megakaryopoiesis and prevent radiation-induced death by annulling thrombocytopenia as well as bone marrow atrophy in the murine model (Satyamitra et al., 2011). Relative to its effect on survival of acutely irradiated mice, the drug has been shown to have a dose reduction factor of 1.32 when administered prophylactically and 1.11 when given following irradiation (Satyamitra et al., 2013). Although this agent has demonstrated efficacy as a radiation countermeasure when tested against acute gamma ray exposures (^60^Co γ-rays), it failed to protect mice against mixed field (γ and neutron) exposures (Cary et al., 2012).

#### Other Agents

##### Metformin

Metformin is a biguanide drug used in the management of type II diabetes (Corcoran and Jacobs, 2020). It controls free radical generation and cellular metabolism, and these effects may be due to the activation of adenosine monophosphate-activated protein kinase. This agent has been demonstrated to have radiomitigative efficacy for acute radiation syndrome (Miller et al., 2014; Xu et al., 2015). Metformin stimulated hematopoietic functions and spleen colony formation when administered *ip* to C3H mice at a dose of 250 mg/kg 24 h post-total body irradiation with 7 Gy (Miller et al., 2014). Spleen colony formation was also shown when this agent was used in combination with other antioxidant agents such as amifostine, captopril, MESNA, or N-acetyl-cysteine (NAC). Metformin administration in combination with these agents significantly increased survival. Metformin was administered 24 h post-irradiation with captopril (200 mg/kg), NAC (400 mg/kg), and MESNA (300 mg/kg). Each of these agents are FDA-approved and have well-characterized safety profiles as well. Metformin alone or in combination with other sulfhydryl agents listed above demonstrated protective efficacy when administered 24 h post-irradiation compared with efficacy for prophylactic amifostine. Metformin at a dose of 250 mg/kg/day orally one day prior to irradiation and 7 days post-irradiation with 4.0 Gy reduced DNA damage and free radical generation in mice. Such changes were linked to an increase in hematopoietic stem cells in bone marrow and stem cell function (Xu et al., 2015).

##### Statins

Statins are structural analogs of inhibitors of 3-hydroxy-3-methylglutarylcoenzyme A, which is a rate-limiting enzyme for the synthesis of cholesterol, and serves to upregulate low-density lipoprotein (LDL). Therefore, these very effective drugs are most commonly used clinically for reducing LDL (Diomede et al., 2001; Gotto, 2003; Grobbee and Bots, 2003) and, consequently, to treat atherosclerosis and hypercholesterolemia (Sizar et al., 2020). In addition, statins are well known to reduce the expression of chemokines. Recruitment of inflammatory cells has also been shown to be reduced by statins in various tissues. Lovastatin treatments of irradiated mice (15 Gy whole-lung irradiation) starting immediately after irradiation or 8 weeks post-irradiation (three times a week) demonstrated a reduction in lung tissue lymphocytes and macrophages. This treatment also improved rates of survival, decreased collagen content, and decreased chemoattractant protein-1 compared to the control group (Williams et al., 2004). Further, the statin treatments appeared to dampen the radiation-induced increases in chemoattractant protein-1 levels.

Simvastatin treatments have been shown to mitigate, to a limited extent, radiation-induced enteric injury, as evidenced by improved structural integrity of the mucosa, reduced neutrophil infiltration (myeloperoxidase-positive cells), decreased thickening of the intestinal wall as well as subserosa, and reduced accumulation of collagen I (Wang et al., 2007). The effect of simvastatin was pronounced for delayed injury as compared to injury that manifests early. Simvastatin treatments ameliorate intestinal damage in thrombomodulin mutant mice. This suggests that the protective effect of this drug is independent of protein C activation. Accordingly, statins, in general, are being considered for clinical evaluation to reduce the side effects of targeted irradiation of the intestine and on other normal tissues as well.

##### Pentoxifylline

Pentoxifylline is a derivative of methyl xanthin and is shown to possess anti-inflammatory, immunomodulating, and antioxidant properties (Ozturk et al., 2004; Hepgül et al., 2010). It is also known to have phosphodiesterase inhibiting activity. It has FDA approval for claudication and associated arterial disease of the limbs (McCarty et al., 2016). It also reduces the risk of radiation injury in lungs of both experimental animals and radiotherapy patients when administered orally (Koh et al., 1995; Rube et al., 2002; Ozturk et al., 2004). When administered thrice a day at a dose of 400 mg/kg to radiotherapy patients, pentoxifylline is helpful in acute and chronic radiation injuries (Ozturk et al., 2004). In preclinical experimental models, pentoxifylline treatment at a dose of 100 mg/kg/day reduced tumor necrosis factor-α protein and mRNA. In a murine model, it raised glutathione levels and inhibited lipid peroxidation after radiation exposure. Lipid peroxidation may be helpful for the radioprotective efficacy of pentoxifylline (Rube et al., 2002; Hepgül et al., 2010).

It has been shown that treatment with pentoxifylline and α-tocopherol has advantageous effects on myocardial fibrosis induced by irradiation (radiation-induced heart disease) as well as beneficial effects on ventricular function in a preclinical, small animal (rat) model (Boerma et al., 2008).

The combination of γ-tocotrienol and pentoxifylline increased bone marrow G-CSF, IL-1α, IL-9, and IL-6 in the murine model. This combination appeared to be effective in modifying the extent of intestinal injury as well as modulating vascular peroxynitrite production following acute irradiation (Berbée et al., 2011). However, survival studies of mutant mice deficient in endothelial nitric oxide synthase demonstrated that endothelial nitric oxide synthase was not needed for protection of these two agents to lethal irradiation. Combined treatments with both agents increased survival over a single treatment of γ-tocotrienol. However, combined treatments appeared not to have reduced GI injury or vascular oxidative stress beyond what was provided by γ-tocotrienol alone. In terms of the radioprotective efficacy of the combined treatment on the hematopoietic system, the treatment course was tested in the murine model of whole-body γ-radiation (Kulkarni et al., 2013). The combination of these two agents was effective when pentoxifylline was administered 15 min prior to irradiation and γ-tocotrienol 24 h before irradiation. The dose of pentoxifylline was 200 mg/kg and the γ-tocotrienol dose was also 200 mg/kg. The dose reduction factor of this combination was 1.5. Hematopoietic recovery was better in the combined treatment group compared to the single treatment (Kulkarni et al., 2013). Mevalonate was used to abrogate the inhibitory effect of γ-tocotrienol on 3-hydroxy-3-methyl-glutaryl-CoA reductase, and calmodulin was used to reverse the inhibitory effects of phosphodiesterase by pentoxifylline. Mevalonate had no effect on the radioprotection of the γ-tocotrienol and pentoxifylline combination. Since calmodulin abrogated the beneficial effects of these two drug combinations, it was suggested that the mechanism of radioprotection by these drugs involves inhibition of phosphodiesterase.

##### Xigris

Xigris (Drotrecogin Alfa - active ingredient in Xigris, a recombinant form of human activated protein C) has FDA approval for the indication of sepsis and acute organ dysfunction with higher risk of mortality (Aneja and Fink, 2007). It is not indicated for sepsis in patients with a lower possibility of mortality (Bernard et al., 2001; Griffin et al., 2006). Findings of a study using the murine model suggest that pharmacologic augmentation of the protein C pathway by recombinant thrombomodulin and activated protein C may offer an approach for the mitigation of radiation-induced tissue injury and lethality (Geiger et al., 2012).

##### Diclofenac Sodium

Diclofenac sodium {2-[(2,6-dichlorophenyl)amino] benzeneacetic acid, monosodium salt} is a derivative of benzeneacetic acid. It is a non-steroidal anti-inflammatory agent and has FDA approval for osteoarthritis, rheumatoid arthritis, and ankylosing spondylitis (US Food and Drug Administration, 2020a). Recently, the FDA approved this drug (diclofenac sodium topical gel, 1%) for non-prescription (OTC, over-the-counter) use for the treatment of arthritic pain. This process is known as switch of prescription (Rx)-to-OTC (Novartis, 2005). Such a prescription drug switch to non-prescription status can be done only after demonstrating that the medication is safe as well effective, and can be used as self-medication based on the description in the drug label.

This agent has demonstrated significant radioprotective efficacy against whole-body 9 Gy irradiation in C57BL/6 mice (Alok et al., 2013). Recently, it has also been shown to reduce formation of dicentric chromosome, γ-H2AX foci, and micronuclei in response to gamma-radiation exposure in peripheral blood lymphocytes of humans. Both pre and post-irradiation treatments demonstrated efficacy, suggesting that it may work to limit radiation-induced clastogenesis when administered either prophylactically, prior to irradiation, or mitigatively, shortly following exposure (Alok and Agrawala, 2020).

##### CpG-ODN

Bacterial DNA is one of the principal pathogen associated molecular patterns (PAMPs). The recognition of PAMPs is mediated through various Toll-like receptor (TLR) members (Underhill and Ozinsky, 2002; Vasselon and Detmers, 2002). The unmethylated CpG motifs occurrence is higher in the genomes of prokaryotes. This is due to the differences in the methylation as well as the use of dinucleotides. (Cardon et al., 1994). The innate immune system detects such unmethylated CpG motifs through TLRs (Hemmi et al., 2000; Takeshita et al., 2001). During an infection, the release of unmethylated CpG-DNA is known as a danger signal. Such a danger signal to the innate immune system generates a defensive immune response for the host (Wagner, 1999). CpG motifs in synthetic oligodeoxynucleotides (ODN) stimulate a response similar to bacterial DNA, and have ODNs have various therapeutic uses (Krieg et al., 1995; Klinman et al., 1996). Such CpG ODNs have US FDA approval as adjuvants for vaccines (Fehér, 2019). The studies with CpG ODN demonstrate that they stimulate innate as well as adaptive immune responses (Klinman, 2004). CpG ODNs interact with TLR-9 and are rapidly internalized by the cells, and they also interact with TLR-9 on the surface of endocytic vesicles (Hemmi et al., 2000; Ishii et al., 2002). Three different classes of CpG ODNs have been characterized: ‘K,’ ‘D,’ and ‘C’ (Klinman, 2004). These agents have been investigated as radiation countermeasures and were found to ameliorate hematopoietic and intestinal injuries induced by radiation exposure in the murine model (Zhang et al., 2011a; Zhang et al., 2011b; Zhang et al., 2013).

##### Auranofin (Ridaura)

Auranofin is an anti-inflammatory, anti-cancer, neuro-protective, and cardioprotective agent (Han et al., 2008; Madeira et al., 2013; Liu et al., 2014; Hu et al., 2018). It inhibits DNA damage-induced apoptosis in the gut by inhibiting acetylation of p53; it also has an anti-inflammatory effect in colitis (Debnath et al., 2012; Nag et al., 2019). It induces mRNA expression of the heme oxygenase-1 enzyme to promote anti-inflammatory action and reduce H_2_O_2_ production to reduce oxidative stress (Madeira et al., 2013). Ridaura is approved for the treatment of rheumatoid arthritis (Nardon et al., 2016). Auranofin has demonstrated radioprotective activity within gut tissue against a low LET (linear energy transfer) clinically relevant dose of radiation in the murine model (Nag et al., 2019).

### Regulatory Agency Approved Drugs Under Redevelopment for Additional Clinical Indications or Currently Being Investigated for Repurposing for DEARE

Exposure to a high dose of irradiation can cause ARS, a serious clinical syndrome carrying elevated mortality risk. Individuals that are fortunate enough to survive ARS often fall subject to a number of secondary, evolving post-exposure disease states, as documented in various mammalian species, and that are characterized by specific, manifest morbidities and their associated increased mortality risks. Therefore, surviving ARS carries additional risks that often rise with time and with often dire consequences. Such long-term, ‘late-effects’ of radiation exposures have been studied extensively over many decades starting from the dawn of the nuclear age, but this complex of diseases has renewed interest (and has been in part relabeled/recharacterized as ‘delayed acute radiation effects’ or DEARE) of late due to the advent of potential preventive treatments (Medhora et al., 2012; Singh and Seed, 2020b). This disease complex, this syndrome, DEARE, expresses as chronic illnesses involving several important organ systems including, but not limited to the lung, kidney, heart, and GI tract (MacVittie, 2015; MacVittie et al., 2019; Unthank et al., 2019). Expression of delayed effects takes several months to years, and ultimately results in multi-organ failure and mortality. The lung from animals with DEARE demonstrates pneumonitis and fibrosis, and the development of countermeasures for such an indication is very important. There are a several drugs under development for DEARE and its subclinical entities.

#### Angiotensin-Converting Enzyme Inhibitor (ACEi) - Captopril

ACEi has been shown to mitigate late-arizing radiation associated injuries in various organs/organ systems prone to manifest such delayed/late effects, e.g., kidney and lung (Moulder et al., 1993a; Geraci et al., 1995; Molteni et al., 2000; Moulder et al., 2007). Captopril is an ACEi containing a sulfhydryl-analog of proline, and it is known to reduce blood pressure. This agent has FDA approval for the indication of hypertension (U.S. Food and Drug Adsministration, 2015). Usually, ACEi has been evaluated for possible radioprotective/radiomitigative properties. Captopril increases kidney function in animal models of radiation injury (Robbins and Hopewell, 1986; Cohen et al., 1992; Moulder et al., 1993b; Robbins and Diz, 2006; Rosen et al., 2015). The agent has been extensively investigated in various animal models of different organ injuries as a result of radiation exposure in several laboratories (Moulder and Cohen, 2007; Ghosh et al., 2009; Davis et al., 2010; Moulder et al., 2011; Kma et al., 2012; Medhora et al., 2012a; Medhora et al., 2014; van der Veen et al., 2015). Captopril has also demonstrated the mitigation of various parameters of radiation-induced lung injury in several animal models (Ward et al., 1988; Ward et al., 1990). It has also been shown to reduce renal failure in patients undergoing radiation therapy (Cohen et al., 2008). Captopril administered with drinking water (140–180 mg/m^2^/day, comparable with clinical dose) has a dose modifying factor of 1.07–1.17 for survival at 80 days. Its dose modifying factor for tachypnea at 42 days after a single dose of X-ray exposure is 1.21–1.35 (Medhora et al., 2012b).

Captopril and perindopril, another ACEi, have been shown to control radiation-induced injury through the recovery of various blood cell components (Charrier et al., 2004; Davis et al., 2010). Such recovery was linked to the improved survival of progenitors. Its action may be partly due to reduction in inflammation (Zakheim et al., 1975) or the transient quiescence of some types of cells (Chisi et al., 2000; Davis et al., 2010). The effects of ACEi drugs on radiation-induced DNA damage have not been demonstrated (Day et al., 2013). Incoming data suggests that administration of low doses of captopril as late as 48 h post-irradiation for 2 weeks improves survival in mice, and such increased survival is also linked with hematopoietic recovery as well as reduction in inflammation (McCart et al., 2019). Captopril, lisinopril (Prinivil), enalapril (Vasotec), and ramipril (Altace) all appear to mitigate radiation-induced nephropathy in rats. Captopril has been shown to be a better mitigator than lisinopril, enalapril, or ramipril. Fosinopril is not an effective radiomitigator for irradiation associated pneumonitis, and it does not mitigate radiation-induced nephropathy (Moulder et al., 2014). These results from a large number of studies demonstrate that captopril is a drug to mitigate radiation injury in humans. In another radiation study of delayed effects, captopril, enalapril, and fosinopril have been shown to increase relative rates of survival against lethal irradiation (Medhora et al., 2014). The use of enalapril in rats at a clinically relevant dose after partial-body irradiation has shown promise for protecting the lungs and kidneys (Cohen et al., 2016).

Captopril has also been evaluated as a countermeasure in a murine model of irradiation plus skin burn (Islam et al., 2015). Results of this study demonstrated that captopril may act in a different way in two types of injuries; namely, irradiation alone and radiation plus a skin burn injury (combined injury). This study also demonstrated that captopril along with an antibiotic may be inappropriate for treating combined injury (Islam et al., 2015).

Recently, a study was performed with ramipril, another ACEi approved by the FDA, investigating the drug’s potential mitigative actions on irradiation associated myelopathy of the cervical spinal cord model using female Sprague Dawley rats (Saager et al., 2020). Contrary to other ACEi, ramipril (more specifically, its active form ramiprilat) crosses the blood brain barrier (BBB) (Nordström et al., 1993). Administration of ramipril reduced the frequency of paralysis at higher photon doses (LINAC - linear accelerator) and also for exposures with high-LET carbon ions, suggesting that ramipril’s effect is independent of the radiation quality (Saager et al., 2020).

#### Surfaxin

Respiratory distress syndrome (RDS) is an important cause of mortality in neonates. Surfaxin (lucinactant) is a synthetic, peptide-containing surfactant approved by the FDA in 2012, and it is used clinically for the prevention of infantile RDS (Piehl and Fernandez-Bustamante, 2012). Because acute radiation-induced lung injury commonly manifests as acute pneumonitis and pulmonary fibrosis, and because surfactant is a well-recognized natural physiologic protectant of lung tissue that is often depleted during various pulmonary pathophysiologic conditions, investigators explored the possibility that therapeutic doses of surfactant (Surfaxin/lucinactant) might well mitigate subsequent evolving lung disease. This concept (mitigative potential of lucinactant) has been tested in a murine model for its mitigative actions on lung injuries. Intranasal administration of KL4 surfactant (lucinactant, 120 mg/kg, twice daily) to C57BL/6 mice after irradiation preserved lung function and reduced lung inflammation and oxidative stress, along with corresponding decreases in total white cell counts, absolute neutrophil counts, and in bronchoalveolar lavage fluids. This agent appeared to be a countermeasure for the mitigation of radiation-induced lung injury (Christofidou-Solomidou et al., 2017).

#### Diethylcarbamazine Citrate

Diethylcarbamazine citrate (DEC) is an antifilarial drug used to treat filariasis (Hawking, 1979). Since the prevalence of lymphatic filariasis is rare in the US, DEC is no longer approved by the FDA, but physicians can obtain this drug from the Center for Disease Control once the patient is confirmed to have the disease based on positive laboratory diagnostic results. In addition, it has anti-fibrotic, antioxidative, anti-inflammatory, and anti-carcinogenic properties (Queto et al., 2010). It mitigates inflammation in the lung because it reduces lipoxygenase, cyclooxygenase enzymes, and nitric oxide in tissue (Queto et al., 2010). DEC effectively reduces the oxidative stress and inflammation in radiation-induced lung injury at a dose of 10 mg/kg in mice. DEC did not show any side effects. However, at a dose of 50 mg/kg or 100 mg/kg, adverse effects were noted (Farzipour et al., 2020). DEC was administered to test animals once a day for 8 days and the treated mice were then irradiated (total-body irradiation, X-rays, 5 Gy) on day 9. The efficacy of DEC was investigated by evaluating oxidative stress by histopathological examination one week after irradiation using lung tissue. Biochemical data revealed increased production of nitric oxide, malonyldialdehyde, and protein carbonyl levels in irradiated animal lungs. In untreated control animals, histopathology examinations revealed acute lung injury along with increased numbers of tissue infiltrating inflammatory cells. DEC treatments prior to radiation exposure appeared to have mitigated the oxidative stress and histological injury within the irradiated animals. Surprisingly, the optimal radioprotective efficacy was seen at 10 mg/kg compared with higher doses. The highest dose of 100 mg/kg did not show protective or anti-inflammatory effects in mice, while the intermediate dose, 50 mg/kg, proved to be somewhat less effective than the lowest tested dose of 10 mg/kg. This study provides suggestive observations indicating that DEC has antioxidant and anti-inflammatory properties at a dose of 10 mg/kg, and that it might be considered for the treatment of radiation-induced lung injury.

### Regulatory Agency Approved Drugs Under Redevelopment for New Indications Related to Cutaneous Injuries or Being Evaluated for Possible Repurposing

Radiation exposures of skin with significantly higher doses of radiation (>15–20 Gy) result in a discrete clinical manifestation characterized by initial erythema, followed by blistering and necrosis. Usually, such necrosis appears 10–30 days after exposure and depends on the extent and quality of irradiation, but in extreme situations, necrosis can appear within 48 h (Peter and Gottlöber, 2002). Peter and Gottlober described the guiding diagnostic and therapeutic principles for individuals with cutaneous radiation injuries (Peter and Gottlöber, 2002). A current strategy, however, employs just two basic steps: first, the initial surgical removal excision of the necrotic tissue in order to prevent recurrence and second, a more recent therapeutic modality that employs treatments with autologous keratinocytes plus allogeneic stem cell administration (Lataillade et al., 2007; Bey et al., 2010). Swine are appropriate for studying radiation-induced cutaneous injury, and the data from NHPs for cutaneous effects are limited. There are several radiation medical countermeasures demonstrating efficacy for the cutaneous injury (Singh and Seed, 2017).

#### Silverlon

Argentum Medical has received FDA approval for multiple indications for Silverlon over a period of more than 20 years. In 2019, it was repurposed for blister injuries caused by sulfur mustard (Argentum Medical, 2019). It is extensively used to manage acute skin wounds and first and second-degree thermal burns. There is interest to repurpose Silverlon burn and wound dressings for large scale scenarios as a result of chemical, thermal, and radiological exposure events (DiCarlo et al., 2018). Silverlon burn dressings are elastic bandages of nylon with incorporated metallic silver. Silverlon burn dressings and Silverlon burn gloves have been extensively used by the US military (Pozza et al., 2014; Aurora et al., 2018).

### Regulatory Agency Approved Drugs Under Redevelopment for New ‘Late Effects’ Indications or Being Evaluated for Possible Repurposing

Exposures to chronic irradiation are either continuous or intermittent and can cause a wide variety of serious injuries, especially those at moderate or low dose rates, that often remain latent for prolonged periods, but evolve with time into potentially fatal diseases. Due to the basic nature of these injuries, often involving radiation-induced changes within genomes of targeted cells within key organ systems of the body, the diseases that eventually manifest are distinct from those disease entities that arise relatively early following acute, intense radiation exposures. We are referring to, of course, a prominent category of radiation-induced late-effects, namely cancer (Rowley, 1985; Seed et al., 1985; Upton, 1985; Baskar et al., 2012; Jargin, 2014; Ozasa et al., 2019; Jargin, 2020). In terms of radiation induction, these late-arizing cancers are often described as being stochastic in nature, with the risk of developing the disease (cancer) following exposure but rather probabilistic relative to the size of the exposed population. This contrasts to the early arising, deterministic-type diseases in which disease risk is directly proportional to the level and intensity of the exposure to the individual. Both acute or chronic exposures to relatively low doses of radiation does not generally cause immediate health problems, but such radiation exposure is a contributing factor for cancer risk to individuals (National Research Council, Biological Effects of Ionizing Radiation, 1990; Khan et al., 2019). It is important to note that a risk that is low for an individual can still result in large numbers of additional cancers in a large population (vs. risk for the population) over an extended period of time.

It is known that combining radiotherapy with chemotherapeutic drugs (e.g., cisplatin) generally improves efficacy for various cancer treatments. However, such a combined treatment strategy comes at a cost due to increased toxicity. Nevertheless, there are opportunities to investigate drug combinations that may increase the benefit of radiotherapy without increasing toxicity inordinately (Khan et al., 2019). Several classes of anti-carcinogenic/anti-mutagenic drugs are being investigated, and a large number of such agents are in preclinical stages of evaluation. The important agents among such drugs are phosphorothioates (amifostine/WR1065) (Grdina et al., 1985; Diamond et al., 1996; Kataoka et al., 1996; Grdina et al., 2000; Grdina et al., 2002). The phosphorothioates are potent radioprotectors when used prior to radiation exposure. As mentioned earlier, such agents have substantial side-effects and toxicity, specifically performance decrement, when administered at doses needed for survival benefits. Investigations from several laboratories over a long period of time have suggested that these drugs can be used post-irradiation and can still preserve a degree of their anti-carcinogenic/anti-mutagenic efficacy, with such effects achieved with much lower and less toxic doses (Grdina et al., 2002; Singh and Seed, 2019). Other agents under development for repurposing include aspirin, vascular endothelial growth factor inhibitors, tumor hypoxia modifiers, immune-checkpoint inhibitors, and DNA double strand break repair modifiers (Khan et al., 2019). Such strategies offer the potential to enhance treatment outcomes for patients with malignancies, principally by decreasing the extent of fractional irradiation, thus reducing radiation-induced side effects. Another benefit would be the decrease in overall cost of treatment. In brief, the success of repurposing such drugs represents “low hanging fruit” in the area of drug development.

### Regulatory Agency Approved Drugs Under Redevelopment or Being Repurposed for New Clinical Indications Related to Radiation Combined Injury

Radiation combined injury is a condition where radiation injury is combined with another insult such as burns, blunt trauma, skin wounds, infection, or hemorrhage. Combined injury is usually more lethal compared with lethality as a result of irradiation alone, or another insult inflicted without irradiation (Anno and Bloom, 2002; Knudson et al., 2002a; Knudson et al., 2002b; DiCarlo et al., 2010). Combined injury accelerates fluid imbalance, cellular injury, circulation failure, myelosuppression, and disorder of organ function leading to multi-organ failure. Regardless of the advancement of knowledge of the radiological injury, there is limited knowledge for therapeutics for combined injury. We do not have an FDA-approved countermeasure for such injuries. Efforts are continuing to develop countermeasures for combined injuries either by developing new countermeasures or repurposing FDA-approved drugs for other indications.

#### Ciprofloxacin

Ciprofloxacin (Cipro) is an FDA-approved fluoroquinolone for the treatment of Gram-negative bacterial infection. It has immunomodulatory and anti-inflammatory potential rather than just being an antibacterial agent (Lahat et al., 2007), and it is also known to enhance neutrophil recovery after bone marrow transplants, suggesting its probable repurposing as a CBRN (chemical, biological, radiological, and nuclear) countermeasure (Imrie et al., 1995). It has been tested in a murine model of radiation combined injury (irradiation and wound). Cipro-treated mice demonstrated enhanced survival when treatment was started 2 h after insult, and such treatment was continued for three weeks (Fukumoto et al., 2014). Its treatment enhanced mouse survival to 80% compared to 35% survival in the control group.

Cipro induced erythropoietin in the kidney and bone morphogenetic protein-4 in macrophages of spleen. Also, its treatment increased CD71^+^ colony-forming erythrocytic progenitors (colony forming unit-erythroid; CFUe) in the spleens of treated mice (Fukumoto et al., 2014). Cipro ameliorated combined injury-induced progressive anemia by day 10 post-irradiation. In another study, Cipro treatment (90 mg/kg, q. d., *po*, after combined injury) reduced pro-inflammatory cytokines and chemokines including IL-6 and KC (keratinocyte-derived chemokine, called IL-8 in humans), and enhanced IL-3 production in B6D2F1/J mice (Fukumoto et al., 2013). Animals treated with Cipro demonstrated a higher repopulation of bone marrow cells, low apoptosis, and autophagy in ileal villi. Systemic bacterial infections were mitigated, along with the mitigation of the IgA production. Cipro treatment protected 100% of mice compared to 80% protection in vehicle-treated mice. This report suggested that Cipro may prove to be a useful therapeutic for combined injury. Cipro has also been reported to enhance recovery from hemorrhagic radiation proctitis in radiotherapy patients (Sahakitrungruang et al., 2011). The study suggests that this rather simple antibiotic treatment is both effective and safe for proctitis induced by radiation-exposure. There was improvement in the extent of rectal bleeding, bowel frequency and urgency, as well as diarrhea. Additionally, Cipro has been investigated in the NHP model at the Armed Forces Radiobiology Research Institute using irradiated animals, but results are not yet published.

In addition to the drugs discussed above, there are several dietary supplements (such as melatonin, available without a prescription as an OTC drug) and agents under the GRAS (generally recognized as safe) category that can be developed much faster compared to other conventional prescription drugs (Williams G. M. et al., 2016).

### Regulatory Agency Approved Drugs Under Redevelopment or Repurposing for Treatments of Patients Subjected to Internally Deposited Radionuclides

Internal contamination with radionuclides remains a threat to civilians and the military alike, as such agents may be internalized through inhalation, ingestion, and exposure to wounds. Internalized radionuclides resulting from either radiological accidents or from a deliberate radiological/nuclear attack would require immediate medical treatment. Current treatment options vary and some carry treatment risks. However, the benefits of these treatments generally outweigh the risks. There are only four FDA-approved agents to either prevent radionuclide uptake or to treat individuals with internalized radionuclides: Prussian Blue (ferric hexacyanoferrate), potassium iodide (KI; ThyroShield), trisodium zinc diethylenetriaminepentaacetate (Zn-DTPA), and trisodium calcium diethylenetriaminepentaacetate (Ca-DTPA) (Centers for Disease Control and Prevention, 2016). These drugs are helpful to block the uptake, dilute, bind, or chelate internalized radionuclides. Apart from the use of KI, there is still scant evidence that the above listed agents provide substantial preventive/therapeutic benefits to victims with internalized radionuclides.

Although a limited number of agents have been FDA-approved, several pharmacologic countermeasures are under development for heavy metal toxicities and perhaps internalized radionuclides as well (Singh et al., 2017a; Singh et al., 2017b; Singh and Seed, 2017). Desferal, deferoxamine mesylate (N-[5-[3-[(5 aminopentyl)hydroxycarbamoyl]propionamido]pentyl]-3-[[5-(Nhydroxyacetamido)pentyl]carbamoyl]propionohydroxamic acid monomethanesul-fonate (salt)) is an FDA-approved iron-chelating agent (Novartis, 2011). Cuprimine (Penicillamine - 3-mercapto-d-valine) is an FDA-approved agent for the indication of Wilson's disease (for removal of excess copper by binding), to reduce cystine excretion, and to treat rheumatoid arthritis (MERCK and Co, 2004). This agent is also being evaluated for the treatment of internalized radionuclides.

## Discussion

Though we are in the age of personalized medicine that attempts to tailor therapies for individuals and specific clinical indications, therapeutic agents that target a large number of diseases regardless of differences in individual patient backgrounds would remain vital for the treatment of a large number of common disorders. Repurposing drugs is defined as ‘finding another novel therapeutic indication for a drug that has been already approved for another indication by a regulatory authority’ (Oprea and Mestres, 2012). This is a novel strategy for finding new therapeutic uses for existing agents that can capitalize on prior investments. This approach is of interest to the drug industry due to the associated monetary benefits to the corporate ‘bottom-line’. Certain clinical applications may be more appropriate for repurposing than others due to the differences in side effects and safety of the drugs involved. These activities are beyond the identification of new targets for existing drugs of another indication. As stated above, despite approximately 90% of drugs failing after progressing to advanced clinical trials and prior to subsequent regulatory approval, it remains encouraging to note that there are still a large number of experimental therapeutic molecules appropriate for human use currently under investigation. There are more than 10,000 approved experimental drugs that can potentially be repurposed. Repurposing is an especially important drug development strategy for rare diseases that require therapeutic options, but have limited resources (Sharlow, 2016).

Radiation induced diseases, especially those that originate from accidental or unwanted exposures, would certainly fall into this category of ‘rare diseases’. Nevertheless, the term ‘rare’ is clearly a relative term in the context of such unwanted radiation exposures; for example, going from a few individuals exposed accidentally during an industrial accident to potentially thousands of individuals exposed as a consequence of a large scale radiological/nuclear attack by terrorists on an urban center. Regardless, there is a real and urgent need to have additional safe and effective pharmaceuticals in storage within the National Strategic Stockpile. It would seem that at present, we (the United States of America) are woefully understocked and ill-prepared for a major radiological/nuclear exposure event. It is our opinion that additional resources need to be allocated by the federal government along with appropriate leadership, and cooperative alliances between the responsible federal agencies and the pharmaceutical industry must be established in order to correct this glaring medical deficit that most certainly impacts national security.

The development of new pharmacologic entities *de novo* for rare diseases is not generally very appealing to large pharmaceutical companies due to the minimal profitability of such endeavors and the associated impact on their financial ‘bottom-lines’. Accordingly, drug repurposing represents an attractive strategy to accelerate the progress of a drug from the laboratory to the clinic, as this pathway bypasses many of the steps of conventional drug development, leading to reduced time and significant cost saving for drug regulatory approval. The examples of repurposing Neupogen, Neulasta, and Leukine as radiomitigators for H-ARS following the FDA Animal Rule demonstrate the advantages of repurposing any widely used drugs that have enormous preclinical and clinical data available, as well as the experience of using the agents in millions of patients over several decades. This should serve as a success story for the drug development of radiation medical countermeasures following the repurposing route (Farese and MacVittie, 2015; National Institute of Allergic and Infectious Diseases, 2015; Singh and Seed, 2018; Gale and Armitage, 2021).

Drug repurposing has been facilitated by the availability of FDA-approved drug libraries, and there are several commercial libraries available for these repurposing efforts (Collins, 2011). However, there are major challenges including regulatory hurdles that need to be tackled (Williams G. M. et al., 2016). There are numerous reasons for failures in the repurposing field which include patent considerations, regulatory considerations, and organizational hurdles in addition to the legal and intellectual property barriers.

With respect to the attempts to repurpose drugs as potential medicinals that might impact public health and overall national security (e.g., CBRN medical countermeasures), strong input from the federal government in active collaboration with the pharmaceutical industry is absolutely essential in order to achieve success in bringing such critically needed medicinals through advanced testing, regulatory approval, and to the shelves of the National Pharmaceutical Stockpiles; and, of course, to the commercial marketplace itself.

Sometimes the repurposing of a drug becomes complicated due to a variety of commercial concerns (and not necessarily ‘public health or national security concerns’); for example, a concern of the developing/sponsoring pharmaceutical organization is that new indications may undermine existing markets of the drug. The sponsoring corporation of a blockbuster pharmaceutical is usually not interested in another indication, especially for rare diseases. Drug sponsoring corporations become wary that during the investigation for repurposing, some information may come to light which can adversely affect the existing market of the drug. Anticipated lower drug prices for the repurposed drug, short patent duration, and overall low return on investment are some of the many reasons that the drug industry tends to be uninterested in repurposing endeavors. Another important aspect is the intellectual property rights (IPR) (Nosengo, 2016). Though some aspects of IPR may no longer be binding or cannot be used, several other patenting/IPR aspects for different dosing, different administration routes, poly-pharmacy approach, and combined usage are available to protect the interest of repurposing. Drug development professionals agree that the perfect candidate for a repurposing program would be a safe and off-patent pharmaceutical agent for which a novel target is known. The efficacious dose of such a drug for a new indication should be within the dose range for an already-approved indication (Oprea and Mestres, 2012).
